# Fluid Intake Monitoring Systems for the Elderly: A Review of the Literature

**DOI:** 10.3390/nu13062092

**Published:** 2021-06-19

**Authors:** Rachel Cohen, Geoff Fernie, Atena Roshan Fekr

**Affiliations:** 1 The Kite Research Institute, Toronto Rehabilitation Institute—University Health Network, Toronto, ON M5G2A2, Canada; geoff.fernie@uhn.ca (G.F.); atena.roshanfekr@uhn.ca (A.R.F.); 2 Institute of Biomedical Engineering, University of Toronto, Toronto, ON M5S3G9, Canada

**Keywords:** drinking monitoring, liquid intake, hydration monitoring, fluid intake detection

## Abstract

Fluid intake monitoring is an essential component in preventing dehydration and overhydration, especially for the senior population. Numerous critical health problems are associated with poor or excessive drinking such as swelling of the brain and heart failure. Real-time systems for monitoring fluid intake will not only measure the exact amount consumed by the users, but could also motivate people to maintain a healthy lifestyle by providing feedback to encourage them to hydrate regularly throughout the day. This paper reviews the most recent solutions to automatic fluid intake monitoring both commercially and in the literature. The available technologies are divided into four categories: wearables, surfaces with embedded sensors, vision- and environmental-based solutions, and smart containers. A detailed performance evaluation was carried out considering detection accuracy, usability and availability. It was observed that the most promising results came from studies that used data fusion from multiple technologies, compared to using an individual technology. The areas that need further research and the challenges for each category are discussed in detail.

## 1. Introduction

Dehydration is a common issue in elderly people that can lead to serious complications and death. Patients admitted to the hospital for dehydration have a 17% chance of mortality in the first 30 days and a 48% chance after 1 year [1]. Even after accounting for confounders, elderly dehydrated patients admitted to the hospital are six times more likely to die than those with normal hydration status [1]. Dehydration is subdivided as hypertonic (water loss, caused by inadequate intake, sweating/evaporation or vomiting), isotonic (salt and water lost proportionally caused often by diarrhea), and hypotonic (salt loss, often caused by diuretics) [2]. Seniors are at a higher risk of hypertonic dehydration for a multitude of reasons surrounding reduced fluid intake and excessive output. The total amount of water in the body reduces by 10–15% in seniors [3,4], which makes them vulnerable to small volume fluctuations [1,5]. The sensation of thirst decreases in seniors [5], while they have more difficulty concentrating urine in the bladder [6,7]. If a healthy young person limits fluid intake, urine output will also decrease; however, this is not the case for elderly people [6]. Older adults are more susceptible to chronic renal problems, causing electrolyte abnormalities; however, these abnormalities can also occur without renal problems [1]. Dehydration can result in confusion, seizures or even death [8]. Medications such as diuretics, which are commonly prescribed for heart failure and some kidney disorders, can cause excessive urination, leading to fluid and salt loss [9]. Additionally, some elderly patients with dementia forget to drink, and some choose not to drink because of a fear of experiencing an incontinent episode or urinating too frequently [10]. Other factors including swallowing difficulties, ambulation difficulties, and dexterity can also lead to dehydration in seniors by lowering liquid intake [8]. 

Monitoring fluid intake/output (fluid cycle) is critical to either diagnose or prevent complications. For example, heart failure patients must track their liquid intake and output volume to ensure they are not retaining liquid in the body [11]. People with chronic kidney stones must track fluid intake to ensure they are sufficiently hydrated to prevent stone formation, and aim for a urine output of 2.5 L per day in adults [12]. Although some studies mentioned that the gold standard to measure hydration level is blood serum/plasma osmolality (P_osm_), this has been highly disputed [13,14,15]. This method is invasive and is impractical for routine measurement. Some researchers argue that though P_osm_ is not adequate to assess chronic dehydration status as it changes constantly, it is best for detecting acute dehydration [16,17]. However, since P_osm_ varies throughout the day and is very dependent on other factors, Armstrong et al. showed that it is inaccurate in reflecting the total body water gains and losses and should not be used in a clinical setting [13]. P_osm_ also can only diagnose the water-loss dehydration and cannot be used to diagnose water-solute-loss [18]. There are also several other common methods to detect and measure dehydration including urine osmolality, urine color, urine specific gravity, weight fluctuation, bioelectrical impedance (BIA), urea creatinine ratio, tongue/mouth dryness, dry mucosa, jugular distention, axillary moistness, sunken eyes, skin moisture and turgor [19,20,21,22,23]. Though some of these methods have been validated in younger subjects, there is a lot of controversy about their effectiveness in the older population [19,20,21,22,23]. Urine biomarkers such as osmolality and specific gravity are commonly used to assess hydration status over 24 h periods. However, this technique has conflicting evidence in younger and elderly populations [18,20,24,25]. Many studies agreed that a single sample of both blood or urine is too variable to be used alone [14,24,26]. In general, older adults have more difficulty concentrating urine in the bladder, thus using urine as a marker is less useful for acute dehydration [16]. Some studies validated urine color as a sign of dehydration, but this can be easily affected by medication, diet, and renal function, so a baseline needs to be determined for each patient [19,27]. Vivanti et al. developed a dehydration screening tool involving 24 h urine sampling, evaluation of physical symptoms and a questionnaire [28]. This tool was slightly modified and tested in [29], which found that including subjective questions, such as thirst, was not useful. However, they found a significant association between laboratory markers and the screening tool scores [29]. In a literature review by Hooper et al., they concluded that from the contradicting evidence in literature, standalone tests including dry mouth, feeling of thirst, heart rate, urine color, urine volume, BIA of intracellular or extracellular water, and fluid intake were not solely able to detect dehydration level in seniors [30]. The authors conducted another study and found that reporting missing drinks between meals, expressing fatigue and BIA resistance at 50 kHz could potentially be useful in identifying dehydration [30]. The same team performed the largest study to date, to our knowledge, to test common dehydration detection methods in 162 elderly patients in a senior home [20]. The findings confirmed that despite contradicting evidence in the literature, specific gravity of urine, urine color, and urine osmolality were not accurate detectors of dehydration in a senior cohort [20]. Urea and creatinine ratio is often used in healthy athletes to assess dehydration, but this can be elevated for a variety of reasons other than dehydration in seniors [16]. In healthy patients, a rapid change in body weight of 3% or more could be a sign of dehydration, but this is heavily influenced by food intake, time of day, clothing weight and accuracy of the scale [19]. Liu et al. used a camera to capture images of the skin to determine skin turgor. However, this technique is not applicable for seniors as they have lower skin turgor resulting from lower collagen levels [31,32]. A dry tongue may be 85% accurate if the dehydration is moderate or severe, but the determination of a dry tongue is subjective and it can be caused by many other factors [19,31]. Additionally, mucosa is often dry in seniors from medical conditions including Alzheimer’s disease, chemotherapy and antidepressants or breathing through the mouth [19,31]. Some physicians also test for an absence of jugular venous distention (determining whether the jugular vein bulges when pressure is applied) to diagnose dehydration, but this is sometimes difficult to visualize and is a subjective method, leading to poor interobserver variability [33]. This was further confirmed by Fortes et al., who conducted hydration assessments on 139 adults after hospitalization [18]. They found that physical signs and urine biomarkers had poor sensitivity [18]. However, they did find that saliva osmolality had moderate sensitivity for both water-loss and water-solute-loss dehydration among elderly [18].

There is no simple, non-invasive and non-controversial method to measure hydration levels in seniors. Many studies emphasized the importance of ensuring proper intake as most seniors have inadequate liquid intake [6,8,20,34], but none implemented a system to monitor fluid intake and output autonomously. There was an overall consensus that if the patient had decreased intake/urination, vomiting, diarrhea, swallowing issues, change in behavior and a higher pulse, they are likely to suspect dehydration [21]. Paulis et al. proposed that a patient-tailored approach considering individual characteristics and environment could be best for diagnosing dehydration, as some seniors present several physical symptoms while others present one or none when dehydrated [21]. 

Elderly patients may initially need extra fluid intake to avoid dehydration, but comorbidities and age-related decline in organ function may enhance the vulnerability to overhydration [35]. Overhydration is when the body has too much fluid and is caused by several conditions that either retain water in the body or cannot remove excess water. For instance, heart failure, kidney failure, liver disease, uncontrolled diabetes and even drinking too much liquid might result in overhydration [36]. People who drink a lot of water to avoid dehydration, including athletes, can develop overhydration as well [36]. Overhydration can lead to hyponatremia, which is when the salt levels are lowered in the body (serum sodium levels < 135 mmol/L) [36]. The consequences of overhydration include heart failure and edema, confusion, high blood pressure, seizure and death [36].

In this paper, we aim to review technologies that are used to monitor oral fluid intake focusing on solutions for the elderly, as dehydration is such a prevalent issue among this group. There are many comprehensive review papers on monitoring food intake and diet; however, to the best of our knowledge, there exists no review paper on monitoring oral fluid intake by recognizing drinking activities. For example, Amft et al. surveyed chewing and swallowing technologies, focusing on food intake [37]. They determined a taxonomy for dietary monitoring technology which included ambient, wearable and implantable devices. Kalantarian et al. [38] reviewed diet monitoring focusing on acoustic, imaging, inertial and manual food diary techniques, while Prioleau et al. [39], Vu et al. [40], and Schiboni and Amft [41] focused on wearable food intake monitoring technologies. Vu et al. [40] analyzed various data filtering and signal processing algorithms and Prioleau et al. outlined common signal processing or machine learning algorithms used on wearable food intake signals [39]. Hassannejad et al. reviewed literature consisting of computer vision and wearable methods for automatic diet monitoring [42], while Stumbo et al. [43] focused on visual approaches only. Recently, Heydarian et al. [44] published a review paper on upper body limb motion sensors to assess eating behavior. This paper discussed future opportunities in the context of dietary assessment and monitoring. These papers focused on eating activity as their primary goal, with only some drinking activities included.

**Figure 1:**
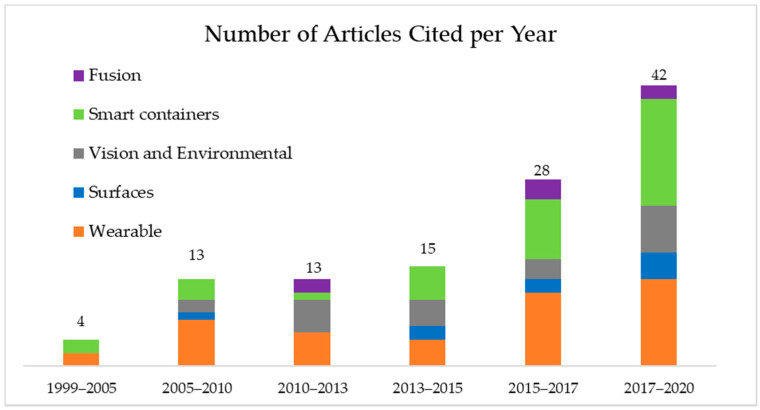
Number of articles reviewed per year.

**Figure 2:**
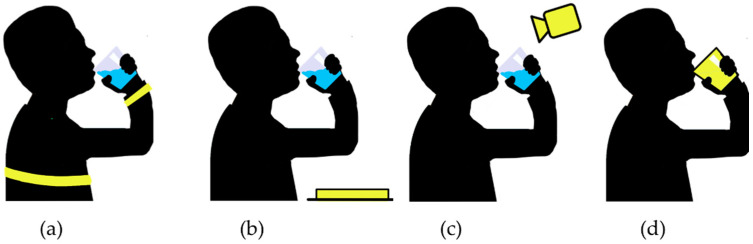
Images of the four reviewed categories including (**a**) wearables, (**b**) surface-based sensors, (**c**) vision and environmental based, and (**d**) smart containers.

In this review, liquid intake monitoring devices are divided into four categories: wearable sensors discussed in Section 3, sensors in surfaces discussed in Section 4, vision- and environmental-based approaches discussed in Section 5 and sensors embedded into cups and bottles discussed in Section 6. Section 7 presents literature that fused multiple sensors from different categories. Finally, Section 8 presents challenges and potential future research for each category, respectively. A breakdown of the approaches in this review is shown in Figure 1. A visualization of these four categories is found in Figure 2.

## 2. Methods

Google Scholar was used to take an initial sample of articles available using broader search terms. Then, Scopus, IEEExplore and Web of Science were employed as the primary databases, using more specific search terms. Search terms were selected for each subsection ensuring that synonyms for each word were also included. The following is a list of search terms included, where the ‘+’ represents “and”, the ‘/’ represents “or”, and the ‘*’ represents any suffix that could be added to the word: smart/intelligent + bottle/cup, liquid intake monitoring, wearable + liquid/drink, vision-based + liquid/hydration/drink, drink + detect*, fusion + hydrate* + elder*/senior, RFID + drink*, radar + liquid/intake, drink* + activity recognition/radar/IR, drink* detection/liquid + radar/IR, liquid + volume+ estimate*/monitor*, food intake + infrared, drink* + activity recognition, liquid+ volume + estimate*/monitor*. 

The search included peer-reviewed studies from book chapters, journals, and conference proceedings. The main search was conducted from August to November 2020. Additionally, keywords from some of the most influential papers were used as search terms. The references and “Cited By” sections of relevant papers were further explored to find related papers. 

**Figure 3:**
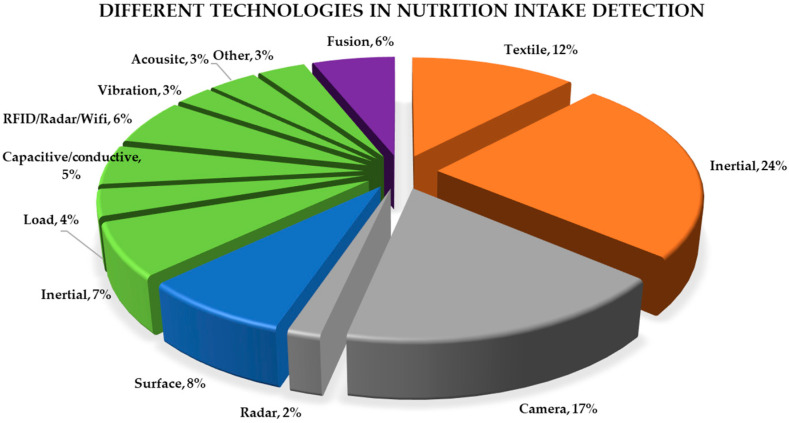
Breakdown of liquid intake monitoring approaches based on the technology used. Orange represents wearables, purple is fusion, green is smart containers, blue is surfaces with embedded sensors, and gray is vision- and environmental-based approaches.

Papers that did not study liquid intake and only studied food intake or other unrelated activities were excluded. Since this review is focused on the elderly population, in the wearable section, we only included literature that used wristbands and textile technology which could be easily worn without affecting the normal daily activity of the subjects. We have excluded devices that were not watch/band or textile based such as throat and ear microphones or ear inertial devices as they are not practical for everyday use. In Section 6, only drinkable containers were included, and monitoring the liquid level of large tanks was excluded. Although this review is focused on the elderly population, studies that used adult subjects were not excluded, as there are too few that only used seniors. The papers were categorized first based on the location of the sensors, for example, “wearable”, “environmental”, “on the bottle”, and “on a surface” and then subcategorized by specific technology used shown in Figure 3. In total, 201 papers were analyzed, and 115 relevant papers were selected for this review. As this is a state-of-the-art review paper, we focused on literature from the past 10 years shown in Figure 1, where more than half of the papers are from 2015 to 2020.

In order to evaluate previous algorithms, we compared different performance metrics such as accuracy and F1-score for drinking detection and the Mean Absolute Percent Error (MAPE) or the Mean Percent Error (MPE) for volume intake estimation. For drinking detection, if there was an unbalanced dataset (meaning there are more data points for one class compared to another), we compared the F1-scores which consider the tradeoff between precision and recall. The MAPE and the MPE reported the standard errors which indicate how much the intake volume estimates differed from the actual values. The MPE shows whether the model underestimates or overestimates, while the MAPE gives a better sense of the true error for each individual intake.

## 3. Wearable Technology

Several wearable techniques are proposed to detect food or liquid intake including inertial measurements of the wrist or body [45,46,47,48,49,50,51,52,53,54,55,56,57,58,59,60,61,62,63,64,65,66,67,68,69,70,71,72,73], or textile-based measurements [74,75,76,77,78,79,80,81,82,83,84,85,86]. The majority of studies analyzed and classified food intake with less focus on liquid intake. This section will review 28 papers using wearable body sensors (mostly in the form of a wristwatch) and 13 papers using textile sensors and exclude throat or ear wearables, as they are not practical for long-term use.

### 3.1. Intertial

Several research papers have used Inertial Measurement Units (IMUs) on the wrist to measure liquid intake. Most previous work focused on intake recognition, but did not attempt to provide an intake volume estimate [45,46,47,48,49]. The wrist-mounted inertial sensors are often used in combination with machine learning (ML) algorithms to detect drinking events. However, some studies used template-matching or threshold-based algorithms [50,51]. For example, Shen et al. used thresholding to segment the events based on the wrist roll values, minimum time between peaks, and velocity [50]. They reported a low sensitivity of 66–75% for drinking detection in an unrestricted setting [50]. Additionally, Eskandari et al. created motion templates using two 3D wrist accelerometers and gyroscopes to differentiate between eating and drinking [51]. Despite having a large dataset of 22,383 intake events, the accuracy of identifying 5 classes (drinking, single hand eating, double hand eating, fork and spoon) was 46%. This low accuracy rate was mainly due to the motion artifact, which is a major problem in inertial signals [51]. In this study, drinking detection did outperform eating with 83% accuracy, and it was concluded that food and drink events had different wrist motion patterns [51]. 

The majority of recent papers used Artificial Intelligence (AI) to detect drinking using wearable devices. Considering single wrist inertial devices, fluid intake classification was performed using Convolutional Neural Networks (CNN) with Long Short-Term Memory (LSTM) networks or LSTM alone [46,52], hierarchical temporal memory (HTM) [48], log regression with Artificial Neural Networks (ANN) [53,54], Random Forest (RF) [47,54], Hidden Markov Methods (HMMs) with k-nearest neighbors (kNN) [55] and HMMs with Gaussian Mixture Modelling (GMM) [56]. Weiss et al. tested several ML algorithms on various Activities of Daily Living (ADL), including but not specific to drinking actions. This included RF, decision trees, instance-based learning, Naïve Bayes and multilayer perceptron algorithm, a type of ANN [57]. The results from the accelerometer signal in the watch significantly outperformed the gyroscope and overall the RF algorithm provided the best results [57]. Additionally, models trained and tested on the same person had an overall higher average accuracy than models validated based on Leave One Subject Out (LOSO) as expected [57].

Several studies failed to have a Null class [48,52,58], which includes general activities rather than only the specified classification labels. Among the papers reviewed, the highest accuracy of >99% was achieved by LSTM for all 5 classes in identifying eating (spoon, fork, knife, hand) and drinking using gyroscope signals from the wrist and waist of the participants [52]. The additional gyroscope on the waste determined whether the person was standing or moving [52]. This paper found that gyroscope data is preferred over accelerometer data and led to a higher accuracy [52]. However, this study did not include a Null class that specifies the “other activities”, meaning that only the predefined gestures were included in the dataset. The inclusion of a Null class can significantly affect the accuracy of the study, so this has been added as an important factor as seen in Table 1.

From the studies including a Null class, Wellnitz et al. achieved an average accuracy of 95.7% for drinking detection using a single IMU on the wrist and 81.4% when placing the IMU on eye glasses [46]. Moreover, classical algorithms including Support Vector Machine (SVM), kNN and RF performed as well as the hybrid CNN-LSTM deep learning model [46]. This study also found that there was a low correlation (0.49) between length of drink and volume consumed across the 41 participants, showing that this is not a reliable way to estimate volume intake [46]. They further attempted to create a ANN architecture by the zero-padded inertial data. Although this new network was superior to random guessing, it was still not accurate to estimate the volume intake [46]. Among papers including a Null class, Anderez et al. achieved the best intake classification results, using a multistep algorithm with a single wrist IMU [54]. They tested binary, 3-class (Null, drinking and eating), and 5-class (Null, drinking, spoon, fork, hand) models. The initial stage of the developed algorithm used a Crossing-based Adaptive Segmentation Technique (CAST) to recognize periods of intake with 100% recall which removed false positives before preforming classification. Then, gesture recognition was applied with either RF (for binary classification) or ANN (for three and five class) algorithms. The system achieved an accuracy of 97.4% for binary classification, 99% for the 3-class and 98.6% for the 5-class classification models. The recall and precision were both approximately 93% for the 3-class, but the system was only tested on a small sample size of 6 subjects [54]. The authors also previously tested a CNN model to recognize dietary gestures including drinking with an accuracy of about 97% [63,64].

Chun et al. focused on detecting drinking using adaptive segmentation with a single commercial wrist sensor with 30 participants drinking from four containers and performing a multitude of ADLs including eating and drinking [65]. They achieved an average precision 90.3% and recall 91% to detect drinking with binary classification [65]. Instead of using the traditional fixed length sliding window, they used an adaptive approach which allowed them match the exact duration of the drinking instances [65]. They found the RF model performed the best with LOSO cross validation, using 45 extracted features [65]. Alternatively in the DrinkWatch, Flutura et al. showed that ML models can be deployed directly on smartwatches to detect drink events in real time [66]. This also had a built-in user feedback system where the classification results could be corrected by the user. However, when running their app on the watch, the battery life was reduced to only 4 h [66]. Gomes and Sousa were able to perform the drinking recognition in real time and could also predict a hand to mouth action 0.7 s before it occurred using a single commercial IMU on the wrist [47]. The RF model was tested both offline and in real-time with an average F1-score of 97% and 85% for drinking recognition, respectively [47]. The data was collected from 12 participants in a lab environment and 5 in a free-living environment. Both scenarios included a Null class of “non-drinking” excluding “eating”. The lower F1-score of the real-time scenario is likely due to more motion artifacts in a real-world environment. A subsequent study by Gomes to evaluate free living conditions also found RF to be the most effective model in detecting fluid intake activity [67]. The accuracy was superior using binary models over multiclass (with eating, drinking, and Null) models [67]. The study obtained an F1-score of 97% for one young participant using the device all day, and 93% average F1-score for 5 others [67]. 

Hamatani et al. and Huang et al. were the only studies that estimated liquid volume intake using wrist IMUs and machine learning [59,60]. Both studies first performed a binary classification, then a gesture spotting (3 sub-classes) and finally intake volume estimation. The gesture spotting sub classified the detected drinking events into micro-activities such as lifting the bottle, sipping and releasing the bottle. Additionally, both studies had a Null class of various activities and various drinking containers which created a dataset of 70 and 20 subjects, correspondingly. Hamatani used a linear regression model in a laboratory setting and in free living conditions to obtain a MAPE of 31.8% and 34.6%, and a MPE of 14% and 15% for intake volume estimation, respectively [59]. This test was limited as the users had to remain relatively still while drinking and they had to place the bottle down before picking it up to take another drink [59]. The drink detection had a precision and recall of 84% and 87%, respectively with an 8-class Conditional Random Fields (CRF—a prediction modelling algorithm often applied in structured prediction.) [59]. They used sip duration, integration of the x-axis and y-axis as features to estimate fluid intake, and found a correlation of R = 0.69, −0.6, and −0.55, respectively [59].

Huang et al. tested several machine learning algorithms [60]. A binary Adaboost algorithm was used for drinking events recognition with 94.42% accuracy, RF was used for gesture spotting (lift, sip, release) with 90% sensitivity and 92% precision, and finally a linear SVM algorithm was used for fluid intake estimation with a 40.11% MAPE [60]. Amft et al. also used a wrist accelerometer to detect drinking events and further attempted to classify the liquid level and container type based on upper body posture [45]. This model could not estimate the accurate amount of liquid intake, as their algorithm only detected three levels of fluid in the container (empty, half full and full) [45]. In a laboratory setting, the algorithm had an average liquid level recognition accuracy of 72% when tested on 9 different containers.

Studies by Junker et al., Amft et al., Moschetti et al. and Ordonez et al. used multiple IMUs on different parts of the body [61,68,69,70]. Amft et al. and Junker et al. failed to include a Null set, and both used HMMs to classify various eating and drinking events [68,69]. Both studies required two IMUs on each arm and Junker added an additional device on the trunk, which is quite cumbersome. Some studies include drinking when performing general ADL classification. For example, Moschetti et al. placed IMUs on three fingers and the back of the hand to test various ADLs including eating and drinking activities [61]. They compared Decision Tree (DT) with an SVM algorithm, and found that the SVM with all 4 IMUs provided the highest accuracy of 91% to classify 9 gestures [61]. Using four IMUs on the fingers is not comfortable nor practical in real life. Using only one IMU on the index finger and wrist was a good balance to compromise accuracy and real-world feasibility, as the system achieved an average accuracy of 89% [61]. The results showed that adding any of the finger IMUs improved the accuracy of the system where the average accuracy using only the wrist IMU was 65.03% [61]. It is worth mentioning that these results are for classifying several gestures and not solely drinking. Ordonez et al. used deep learning on two existing datasets where the subjects had either 7 IMUs and 12 accelerometers (placed across arms, trunk, legs and feet) or 10 accelerometers on each arm to classify ADLs including drinking [70]. They achieved an F1-score of 90% using 8-layer convolutional and LSTM layers to classify “drinking coffee” which might take longer relative to other activities in the dataset [70]. Using their 8-layer algorithm, they achieved superior results compared to the baseline CNN models. 

Merck and Mirtchouk et al. performed several studies on liquid intake recognition using IMUs on both wrists and Google Glasses to get inertial head movement. They reported low recall rates (44–47%) throughout three studies [71,72,73]. In one study, the amount of fluid intake was estimated by a 47.2% Mean Absolute Error (MAE). However, the model was not improved by this additional parameter, but provided the best results when combining both audio and motion data from the glasses.

### 3.2. Textile

Textile applications can provide more information than inertial sensors, including chewing and swallowing detection. However, they are less practical and are often incorporated into shirts in the form of a turtleneck. In this section, there are two main textiles: (1) in the form of collars/bands around the neck, containing electrodes detecting swallows, and (2) using Respiratory Inductance Plethysmography (RIP). When one swallows, there is an apneic event in the respiratory signal captured by RIP. This method detects swallowing based on a continuous measurement of respiration using plethysmography. 

Cheng et al. first used textile-based electrodes integrated into a turtleneck shirt to measure the changes in capacitance in the throat [74]. This was used to recognize chewing, swallowing, speaking and sighing in different head positions, while sitting or walking [74]. Although the authors claimed that the proposed textile approach did not need direct skin contact or excessive fixation to the body, large amount of data was lost in their initial tests. The overall classification accuracy using a threshold-based algorithm was achieved 77% when sitting and 69% when walking. Under sitting conditions, the swallowing liquid action had a classification precision of 60% and recall of 80% when tested on 3 subjects. The neckband system was further validated on 3 subjects for 3 days [75]. They reported a high rate of false positives (136 FP for only 64 true events) considering their small sample size [75]. Zhang et al. investigated a generic fabric to place bio-impedance electrodes and resistive pressure sensors [76]. The fabric was used in the seam of a men’s dress-shirt and was invisible and unobtrusive [76]. This study was only a pilot study with three subjects and they did not report any accuracy values. However, it was found that fusing the pressure and bio-impedance data can improve the results. Amft and Tröster combined surface EMG and a microphone in the collar of a shirt. EMG signals were able to detect the swallowing events, but they did not provide useful information about the volume and viscosity of the swallow [77]. They attempted to classify low and high volume size and low and high viscosity of swallows. They achieved an accuracy of 73% with Linear Discriminant Analysis (LDA) and NB and 75% with kNN. Using three classes (low, medium, high) provided very poor results and was excluded. Subsequently, they could obtain a swallow detection accuracy of 70% [78]. These studies both had four and five subjects, respectively. Farooq et al. detected food and liquid intake using electroglottography (EGG) in a necklace [79]. The EGG mounted on the neck collar monitors the electrical impedance of the larynx to detect swallowing with a detection rate of 89.7% for females and 90.3% for males [79]. EGG signal was found to be superior compared to a throat microphone when tested on 30 individuals [79].

#### Respiratory Inductance Plethysmography (RIP)

Moreau Gaudry et al. detected swallowing events in elderly patients by an elasticized jacket which measured chest movement continuously to obtain the respiration signal, known as an RIP [80]. The accuracy of the system was given by the area under the receiver operating characteristic (ROC) curve, and was greater than 0.9 when tested on 14 subjects [80]. Dong and Biswas expanded this idea with a chest piezo respiration belt to detect pauses in breathing that occur when one is swallowing [62,81,82,83,84,85]. This belt had no contact with the skin and converted tension from chest motion into respiration signals. They first used template matching to classify normal breathing, swallowing while exhaling and swallowing while inhaling [81]. This template-matching algorithm was tested only on three subjects with their own customized templates. This method had an average true positive rate of 92.5% and false positive rate of 3.3%. They also used SVM to perform the same classification with an average recall and false positive rate of about 98.1% and 0.4%, correspondingly [82]. Using time or frequency domain features provided similar classification results. They also tested SVM to differentiate food and drink swallows in 3 subjects, obtaining an average accuracy of 81.3% [77]. In another study, they compared the feature matching and machine learning methods on seven subjects and found that the DT model provides better outcomes compared to the template matching (96.6% recall vs. 82.1%) [84]. In addition, they found that body movements did not noticeably change the breathing signal and the algorithm could correctly classify “spontaneous swallows”, i.e., swallows as a result of saliva only, as non-drinks [84]. This study was later refined to test food intake detection using a two-step SVM with HMM [85]. They tested 6 healthy young adults with no swallowing difficulties and obtained a precision of 75% and recall of 86% on solid food swallows. The combination of SVM and HMM gave better results compared to using SVM alone. They proposed a 3-stage SVM and HMM hierarchical classifier: first detect normal breathing or inhale/exhale swallowing, then detect talking or swallowing food, and finally categorize the swallow into liquid or solid intake [62]. For this experiment, the user had to wear two RIP belts and a wrist accelerometer to aid in determining the meal duration and frequency [62]. The F1-score was improved from 68% to 83% in the second stage when including the hand gesture features; however, these had no effect on the first and third stages [62]. The F1-score for each stage ranged from 82% to 87.2% [62]. Tatulli et al. applied RIP for swallow detection on five subjects using diverse food intake [86]. The method was similar to Dong and Biswas but used quadratic discriminant analysis as the classifier [86]. The overall accuracy was 79%, which was improved further to 86% after using an EGG signal to remove speaking artifact [86]. All reviewed articles in this paper used the RIP signals to detect the swallow events and to our knowledge, RIP technology was not used to estimate the amount of food or drink consumed. RIP belts do not need direct skin contact and can be incorporated into a shirt comfortably. Table 1 summarizes the top studies that used wearable technology for fluid intake detection.

## 4. Surfaces

Tables or table mats embedded with sensors have been used to monitor dietary intake [87,88,89,90,91,92,93,94,95]. Table sensors are used for both drinking and food monitoring, while coaster solutions targeted drink detection specifically. All these solutions used load cell, force-sensing resistors, or pressure mats to measure weight and its distribution.

Chang et al. created a smart table with weight sensors to determine the amount consumed and RFID sensors to label each food [87]. This method provided 80% accuracy detecting eating and various transferring of food events, but was not practical as every food needed an RFID tag [87]. The weight recognition accuracy of the system was 82.62%. The sensors on the table had a very low spatial resolution and all food had to be placed in specific areas on the table to be read by the load cells, properly [87]. Zhou et al. developed a smart table cloth consisting of a textile matrix of pressure sensors (1 cm^2^ resolution) and commercial force sensor resistors on each corner [88,89]. Five subjects tested 40 meals with drinks to recognize different movements of cutting, scooping, stirring, and picking up drinks with 90% recognition accuracy using a subject dependent 7-class DT algorithm [89]. To spot the actions in the continuous data stream, the same algorithm was used and an average F1-score of 87.06% and 71.35% for subject dependent and independent were found, correspondingly. Although they used a high-resolution pressure sensor matrix, the intake weight estimation had a RMSE of 16% [89]. The final system provided high resolution but low weight accuracy. Haarman et al. embedded 199 5 kg load cells in a table for high resolution and tested 4 users eating pie and drinking [90]. Weight measurement errors ranged from 0.3 to 3.6 g, or 0.25% in the pilot study [90]. Mattfeld et al. created a tray with load cells and monitored 271 subjects eating and drinking unrestrictedly in a cafeteria [91]. The algorithm detected the weight of each bite but weighed only 39% of all food and drink bites due to the instability of the scale [91]. Eating and drinking were discriminated using a weight curve with 1 false positive for every 10 bites [91]. Watanabe et al. proposed a portable sensor sheet, similar to a place mat, to monitor food and drink consumption during mealtimes at nursing homes [92]. The system comprised a gel sheet between eight pressure sensors and an acrylic hard board, and was tested by having two caregivers feed two volunteers as if they were in a nursing home setting [92]. The maximum error for food intake volume was 49 g, with an error of 9 g for drink estimation, but this study had a very small sample size of only 2 meals [92]. Lessel et al. developed a coaster using a load cell to track liquid intake and an LED for feedback to remind the user to drink [93]. After 3 weeks with 20 subjects, they found that most subjects reached their liquid intake goals, but they lost some data due to Bluetooth transmission issues and movement of the coaster [93]. Chan and Scaer had a very similar design of a coaster but did not test it on human subjects [94]. Plecher et al. placed force-sensing resistors in a base to measure liquid intake volume [95]. This design is bulky and not transportable, though works in a seniors home to inform the caregiver that the elderly person is drinking [95]. All these methods need the user to place their meal/drinks in a specific area to be able to measure the volume intake or to detect the drinking action. Therefore, for an elderly population, this might not be a practical solution as they might forget easily to put the container in that specific location. Table 2 summarizes the top four studies that used surface technology for fluid intake detection.

## 5. Vision- and Environmental-Based Methods

### 5.1. Cameras

Vision-based approaches used cameras and computer vision algorithms or deep learning to detect drinking activity. Many of the studies in this field used Microsoft Kinect which calculates depth and takes RGB images. Chua et al. applied a Haar-like deep learning feature algorithm on images to detect a hand grasping a cup [96]. This network was only trained with images and did not capture drink intakes [96]. Subsequently, they used a Microsoft Kinect placed in front of the subject to detect various hand postures during the drinking activity [97]. They focused on using the depth information exclusively to eliminate privacy concerns. Drinking events were classified with 89% accuracy using a Dynamic Time Warping (DTW) [97]. Kassim et al. and Cunha et al. also used a Kinect to monitor wrist joint motion and find the number of bites and drinks consumed [98,99]. They used a single frontal view, which can lead to occlusion issues [99]. Burgess et al. monitored fluid intake using a Kinect and a NB Classification, and tested different locations of the device [100]. This study only tested a single subject. They concluded that when the Kinect is placed on the right side of the subject, there was more obstruction detecting fluid intake [100]. Cippitelli used a top down Kinect view to prevent occlusion and combined RGB and depth data to monitor food and drink intake [101]. When testing 35 adults, they achieved a 98.3% average drink detection accuracy using an adapted Self-Organized Map algorithm, a type of ANN algorithm, to detect the human gestures on the depth map, and processing the RGB frame to detect the presence of a glass [101]. Iosifidis et al. created 3D volumes from frames in food and drink intake videos and then performed Fuzzy Vector Quantization and LDA [102]. In low dimensional discriminant subspaces, the activity classes are linearly separable, with a classification rate of 93.3% [102]. 

Some reviewed papers performed general activity monitoring and included drinking as one of their main category. Cebanov et al. detected hand position of seniors while drinking in standing and sitting positions using the built-in skeletal tracking and gesture detection algorithm on images data from the Kinect camera. They obtained a 70% drinking detection accuracy [103]. To protect privacy and to operate in any lighting condition, they only used the IR and depth streams [103]. In addition, they estimated the liquid intake volume by assuming that each intake sip was constantly 100 mL, leading to a poor estimate that was not validated [103]. Chang et al. used deep learning model trained with video and depth stream from a Kinect camera to classify several types of human activities, including drinking [104]. An average accuracy of 96.4% was achieved when combining color, depth and optical flow in a CNN algorithm. When using two Kinect sensors, Brena and Nava achieved superior results classifying four to six activities in a meeting room including drinking water [105]. Using a kNN algorithm, they achieved an average accuracy above 95.4% [105]. This was specifically tested on people seated, and the use of two Kinects aided with occlusion problems. 

Several researchers have developed wearable cameras to monitor food or drink intake, but the majority focused on food [106,107,108,109,110]. Two different studies which used the Microsoft SenseCam wearable necklace camera lost 28–29% of their data due to absent images, blurry/dark images, not wearing the camera, or not wearing the camera properly despite clear instructions to the participants [106,107]. Many studies used images from smartphones to classify food and drinks and determine volume type, but the user had to manually take pictures with a smartphone before and after each meal [111,112,113,114,115]. 

### 5.2. Radar

Although several papers used radar for activity recognition, few had recognized liquid intake. A single paper by Shah and Fioranelli was reviewed in this section which captured human data using Frequency-Modulated Continuous Wave radar (FMCW) to perform activity recognition of six human motions in four geographical locations, including drinking water [116]. SVM, kNN and GoogleNet were tested and the average accuracy was reported between 74.7% and 78.25% [117,118]—Table 3 summarizes the top five studies that used vision-based technology for fluid intake detection.

## 6. Smart Containers

This section describes current research and commercial containers that monitor liquid intake. Liquid level monitoring in smart water bottles is divided into several methods: load and pressure, inertial, capacitive and conductive, radar and WiFi, vibration, acoustic and other. Kreutzer et al. stated that for elderly people, the use of radar, guided microwave, ultrasonic, and flow sensors to detect drinking activity need a lid on top which could impeded the drinking and cleaning process [117]. They claimed that using conductivity, capacity, and load cells were the most practical solutions [117]. Plecher et al. provided a list of requirements for elderly smart bottles/cups such as hygiene, usage of their own cup, and safety [95]. They stated that sensors that are in contact with the liquid are usually less hygienic, likely not dishwasher safe and in some cases less safe [95]. They also stated that if the seniors can select their own cup, they will be more compliant and not feel stigmatized [95]. The selected sensor must be ergonomic so the user does not drop and break it [95].

### 6.1. Inertial

**Figure 4:**
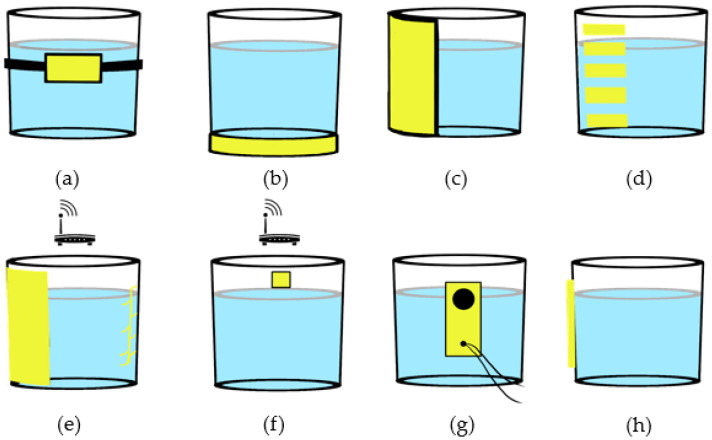
Schematic diagram of various sensor layouts for each smart container category, namely (**a**) inertial [120,121,122,123,124], (**b**) load and pressure [125], (**c**) capacitive [126], (**d**) conductive [127], (**e**) Wi-Fi [128], (**f**) vibration [129], (**g**) acoustic [130], (**h**) and other level sensor [131].

Inertial methods used accelerometers and gyroscopes to determine the orientation of the bottle. This involves placing an IMU on the outside of the bottle. Based on the orientation and duration of the event, the volume of each sip can be estimated. It is also possible to detect whether the contents have been drunk, spilled or poured out. Gellerson et al. was the first to place an IMU in a mug with a temperature sensor to determine whether hot liquids were present, but they did not test it with human subjects [118]. Liu et al. placed an IMU in the base of a 3D-printed cup to detect drinking in every day settings, but they did not estimate intake volume [119]. They found that using kNN to classify drink events achieved F1-score of 89.92% for detecting a drink event within a window and 85.88% to detect the exact frame in 11 participants. Dong et al. and Griffith et al. placed an elastic band with an accelerometer around a water bottle to estimate volume intake and fill ratio (fill level as a percentage of the height of the container), shown in the schematic in Figure 4a [120,121,122,123,124]. They obtained an overall MAPE of greater than 7.64% for the fill ratio and 19.49% per sip using machine learning [120,121,122]. In addition, they found that using the fill ratio instead of volume intake had less inter-subject variability. 

Due to large potential errors, the IMU is often combined with other liquid level detection methods such as capacitive or load cells [125,132,133]. The Playful bottle by Chiu et al. combined the data from an accelerometer and a phone camera attached to a clear plastic cup [134]. This method obtained 96–98% drinking detection accuracy and provided games to encourage hydration [134]. The proposed design creates a bulky and heavy device and it requires a phone placed against a clear cup with clear liquid to determine the volume [134]. 

### 6.2. Load and Pressure

Load cells have been used to monitor changes in weight accurately, but they were not able to detect whether the drinking action was performed or the liquid spilled or poured out. That is why they are often combined with other technologies, e.g., IMUs [125,132,133]. Another limitation is that the container needs to be on a surface to measure the weight and must be calibrated for each container, separately. Zimmerman et al. used strain gauge load cells in the base (demonstrated in Figure 4b) and an IMU in a smart cup holder and tested the design with patients in a nursing home [125]. The holder had a volume intake estimation accuracy of 2 mL. This proposed cup holder was able to increase the liquid consumption from 1.9 to 4.9 L over a 5 month experiment by using feedback sent to the nurses via cellphone [125]. Some commercial products such as the H2OPal also used load cells [132]. Pressure-based approaches have been used to monitor liquid level in industrial tanks but have not been applied to smart bottles. Wang et al. developed a container with a plastic pipe in the middle and a load cell to measure buoyancy force under the pipe [135]. This could be incorporated as the straw of the bottle and had a water level resolution of 1 mm.

### 6.3. Capacitance and Conductivity

Capacitive sensing is often used to estimate the liquid levels in industrial tanks. This method usually requires the sensors to be in direct contact with the liquid [130], which can cause issues with corrosion or the liquid might leak at the electrode liquid interface [136]. Additionally, there is a tradeoff between the number of sensors and the accuracy of liquid level detection. The accuracy is often higher when the entire container surface is covered by the sensors, as the sensors can only discretely determine the liquid level [130]. The capacitive or conductive sensors most often are in the form of stickers placed at discrete levels on the cup wall, either inside or outside of the container. Dietz et al., Kreutzer et al. and Geethamani et al. developed contactless capacitance methods, where the sensors are placed around a large portion of the bottle, not in direct contact with the liquid [126,136,137]. Geethamani et al. did not provide any evaluation results of their system [137]. In the study by Dietz et al., they combined an RFID sensor with the capacitive sensor to locate the cup in a restaurant setting [136]. The drinking container was divided into 16 levels of liquid. They stated in the paper that the accuracy varied depending on whether the vessel was placed on a table, but they did not report any accuracy values [136]. Kreutzer et al. obtained an average error of 3–6% for estimating the liquid level with contactless capacitance sensors placed on the outside of the container similar to Figure 4c [126]. They found that the accuracy was affected by the temperature of the liquid and the location of the user’s hands relative to the sensors [126]. It is also possible to place capacitive sensors on the outside walls or bottom of a cup, as done by Fan et al. in the form of electric tape, similar to Figure 4d but on the outside of the cup [138]. This method does not contact the liquid inside and obtained a correlation coefficient of 0.98, a relative absolute error of less than 16% for all material types tested [138]. To mitigate interferences caused by hands touching the sensor, they added a 3D-printed case, which made the device bulkier [138].

Kreutzer et al. developed a smart cup with an embedded conductive sensor inside the inner wall to monitor the liquid level, also similar to Figure 4d [117]. Various tests were performed to validate their volume level estimation technique. The tests included liquid put in/removed, different beverages, temperatures, various placements of conductive sensors, and cleaning methods [117]. In the preliminary results, it was able to detect liquid levels at all temperatures, but had difficulty in measuring some types of liquid such as those with large remnants at the bottom of the bottle like milk suds [117]. Bobin et al. also used five conductive electrode sensors placed vertically on the inner wall of the container, as depicted in Figure 4d, to detect liquid level, along with an IMU to capture the stability of the motion for stroke rehabilitation [127]. Using a 5-class SVM (including sitting, standing, walking, stairs and drinking), the overall accuracy of the system was 94.33% and a drink class accuracy of 96.98 [127]. 

### 6.4. RFID, Radar and Wi-Fi

RFID can measure liquid level because the signal strength (RSS) and phase are impacted by the volume of liquid [139]. Some papers used RFID to detect drinking events [140,141], empty cups [142] or to identify liquid types since the RSS and phase are also affected by different liquid components (for example, Pepsi and Coke) [143]. Jayatilaka used RFID attached to the bottom of a cup to detect drinking events (binary classification) in young and old subjects with an F1-score of 87% from SVM and 79% from RF [140]. Kreutzer et al. tested the RFID and conductivity to detect liquid level, and achieved a resolution of less than 25 mL per measurement in their preliminary trials [117,139]. The RFID was also placed on the bottom of the cup, and the conductivity sensor attached on the inner wall. The range of most RFID tags is very limited and therefore it is not possible to measure the signal, continuously [139]. In other words, since the RFID is passive, it requires very close proximity to the sensor; otherwise, the RFID readers consume relatively large amounts of power to transmit the data continuously [139].

LiveTag used passive metal tags placed on the container which were remotely detected by WiFi receiver, as seen in Figure 4e [128]. The liquid level was measured by vertically arranged tags with 90% accuracy if the vessel was within 4.8 m of the router [128]. This study had limitations such as: the measurement was not continuous, the container could not be metal, and the container could not be in motion [128]. It is also important to note that liquid monitoring was just one application of their system and not the main focus.

### 6.5. Vibration

Vibration methods determine liquid level by detecting the resonant frequency. Ren et al. attached a low-cost, small transducer to the outer surface of the container, as seen in Figure 4f. This emitted a vibration through the container, which affected the WiFi signals [129]. The phase changes in Wi-Fi signals helps in extracting the resonance frequency of the liquid to determine the level [129]. This method achieved an overall accuracy of 97% when measuring the liquid level continuously with a curve fitting technique and found that 90% of all measurements had an error of less than 6%. This paper also obtained an average F1-score of 96.8% with SVM to classify the liquid level to the nearest marker (10 classes/levels). The device worked in non-line-of-sight trials with different liquid types, container types, angles and distances, but had high power consumption [129]. Nakagawa et al. used a piezoelectric vibrator and measured the amount of mm-waves absorbed by the liquid with a Doppler sensor outside the bottle [144], and Ryu et al. applied a piezoelectric receiver inside the bottle to measure the resonance frequency [145], though neither evaluated the accuracy of their systems. 

### 6.6. Acoustic

Acoustic sensors can also measure liquid volume, by placing the sensor on the outside of the container, as in Figure 4g [129,130]. Tommy et al. proposed a bottle with an ultrasonic distance sensor to monitor liquid level, an accelerometer to monitor the bottle cap position, and temperature and humidity sensors to provide recommendations [146]. The SmartOne device proposed by Vithanage et al. combined a humidity sensor to detect whether a mouth was close, an IMU to detect the drinking pattern, and an ultrasound sensor to measure the volume in the container [147]. It also had a temperature sensor to give accurate intake recommendations, and a pH and turbidity sensor to monitor the quality of the liquid [147]. Wijanarko et al. used ultrasound and ambient temperature to measure the amount consumed and recommend if the user should drink a small, medium, or larger amount of liquids using a Fuzzy Logic algorithm [148]. None of the aforementioned studies tested the system accuracies. Fan et al. acoustically excited a container by outputting a probing signal and at the same time recording the impulse response to determine the content level in the container [130]. This system, as shown in Figure 4g, can be applied to any container in its original packaging including food, but does not work with deformable containers, such as soft plastics [130]. The system obtained an F1-score of 96.9% to 97% for all 19 containers tested [130].

### 6.7. Other

Float sensors have been used in industrial applications to measure liquid level. Pankajavalli et al. used a water float sensor based on a magnetic field that contacts the water and sends a text message when the level is low and high, but this technique was not tested by human participants [149]. Akbar and Oktivasari used float sensors based on the Hall Effect concept to measure the amount of water consumed [131]. This device can provide the amount of daily water required based on a user’s profile [131]. The system had an average error of 1.1% with a maximum error of 2% [131]. This involved placing a sensor on the outside of wall of the container, shown in Figure 4h. 

Ayoola et al. developed a smart cup for heart failure patients to inform nurses of hydration status, but the user needs to manually press a button to register drinks [150]. They did not test if the patient’s behavior changed after the nurse’s recommendations [150]. Lester et al. classified various liquids in a cup using an ion selective electrical pH sensor, conductivity sensors, and optical methods using light [151]. They did not determine the liquid intake amount, but were able to recognize 68 different drinks with 79.4% accuracy [151]. Table 4 summarizes the top eight studies that used smart container for fluid level detection.

### 6.8. Commercial

**Figure 5:**
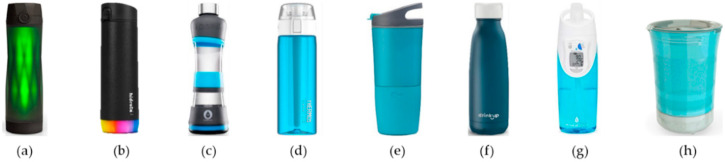
Images of analyzed commercial bottles: (**a**) HidrateSpark 3 [153], (**b**) Hidrate Spark Steel [154], (**c**) H2OPal [132], (**d**) Thermos Smart Lid [155], (**e**) Ozmo Active [156], (**f**) DrinkUp [158], (**g**) HydraCoach [159], and (**h**) Droplet Tumbler [160].

Commercial smart bottles and mugs are becoming more popular especially among active younger people. The commercial water bottle HidrateSpark used the capacitive and IMU sensors into the straw to estimate the volume by an accuracy of 97% (Figure 5a) [152,153]. The recent version, the HidrateSpark Steel (Figure 5b), has all the sensors in the bottom of the device and has the option to have a straw or not [154]. Both versions connect to your phone via Bluetooth and LEDs in the bottle light up to remind the user to drink throughout the day [147,149]. The H2O Pal (Figure 5c) uses a load cell and IMU sensor in the base of the device, and any regular water bottle can be inserted as long as it has a similar size [132]. This device is not rechargeable but has a 6 month battery life and can store data both offline and online [132]. Additionally, the device must be placed on a surface after each drink [132]. The Thermos Smart Lid (Figure 5d) contains the temperature and liquid level sensor in the lid, but cannot withstand hot liquids and must be upright to perform measurements [155]. The Ozmo Active Smart Bottle (Figure 5e) can differentiate coffee and water and tracks both intakes separately and continuously in the app [156]. The Java+ version can also regulate the temperature of the liquid and heat or cool it to a desired temperature [157]. The DrinKup bottle uses ultrasonic sensors placed in the lid and displays the volume intake both on the lid and in the app [158]. It looks identical to a regular water bottle as shown in Figure 5f and can store data offline [158]. This bottle will inform the user if the water is “stale” (has not been replaced in 24 h) and can be used with hot or cold liquids [158]. The HydraCoach (Figure 5g) logs water using an impeller placed in the straw of the device [159]. The logs are only stored locally and displayed on the build in LCD screen [159]. The Droplet cup (Figure 5h) is specifically designed for seniors to be lightweight and ergonomic [160]. The cup and mug version can use the same base, and custom audio recordings remind the senior to drink regularly [160]. Plecher et al. compared the practicality of different smart bottles in terms of key feature, liquid leveling, user interaction and suitability for elderly people [95]. However, some of the bottles included in this study were no longer available to purchase [95]. Figure 5 and Table 5 show the eight commercial bottles described in this study.

## 7. Fusion

Several researchers attempted to combine data from multiple sources to obtain more accurate results. Hondori et al. fused data from a Microsoft Kinect depth camera and inertial sensors placed on utensils and cups [161]. This was a pilot study with one participant and was not clinically tested, but preliminary results showed that position, displacement and acceleration of the arm can be captured properly [161]. Additionally, this study did not attempt to classify the movements to detect drinking actions [161]. Troost et al. fused wrist inertial data with camera data to improve eating and drinking recognition [162]. However, they concluded that the dataset is too small to evaluate the performance [162]. Soubam et al. combined a load cell in a cup with an accelerometer on the wrist to estimate both the volume intake and if the liquid was spilled or dumped [133]. This setup was tested on 3 different containers with 6 different liquid types to obtain a drink detection accuracy of 95.97% with RF and volume intake accuracy of 98.34% [133]. Each container needed to be recalibrated every time, which was an issue with disposable containers. Seiderer et al. combined a weight scale, a smartwatch, and a smartphone to monitor food and drink intake [163]. The author did not report any evaluation results.

Jovanov et al. implemented and compared two systems to detect drinks and measure the amount [164]. The first method used a capacitive sensor that detects touches of the bottle and monitors PPG. The second method combined two accelerometers, one on the bottle and one as a wearable around the user’s wrist [164]. This product was not tested with human subjects but preliminary tests showed that both systems could be viable depending on the application. 

Garcia-Constantino et al. fused data from a wearable accelerometer, contact sensors placed around a kitchen (on kettles, cups, doors, cupboards, containers) and thermal sensors placed on the ceiling [165]. This study involved 30 participants all performing the same action of entering the kitchen, preparing a hot drink, consuming the drink and leaving. No other activities were recorded (no Null class) [165]. Drinking actions were detected with 95% accuracy, but the overall system accuracy for 4 classes was only 73.51% since entering and exiting were often poorly classified [165]. The HydraDoctor by Du et al. uses a commercial wrist accelerometer to first detect drink events and then triggers smart eye glasses (Google Glasses) to record a video [166]. The video is used to classify the type of container and beverage from a database [166]. With 11 subjects and 6 types of liquid, the system had an accuracy of 85% drink detection and could classify the liquid type with 84.3% accuracy [166]. This system claimed that it can detect the amount consumed, but this was not tested in the study [166].

## 8. Discussion and Overview

The analysis of the literature showed that the majority of studies applied machine learning to perform classification as it outperforms template-matching and threshold-based techniques. Although most of the studies performed the classification offline, there exist recent papers that attempted to use the classification in real time or on the watch directly. However, more research is needed to improve the accuracy and optimize the power consumption. Additionally, though many papers claimed to target seniors, only five of the reviewed papers tested the systems with elderly subjects and only one commercial water bottle targeted seniors. Hydration status in seniors is a recognized prevalent issue, but technological solutions to monitor fluid intake in this demographic are not heavily explored in research.

### 8.1. Wearables

The analysis showed that the majority of wearable devices provided useful information to detect drinking activity, are scalable and widely available commercially. In addition, this method is not limited by the type of container or location (i.e., can be in used public). There is also no risk of obstruction or occlusion and the device can be worn easily on the wrist without disturbing the user. However, wearables have a high rate of false positives and do not detect drinks when drinking from a straw or if using the opposite hand that does not have the device. Additionally, some elderly people are not compliant with wearable devices and do not want to wear the devices. To classify drinking activities, RF models were among the superior models. To date, wrist wearables have not accurately estimated fluid intake volume. However, there has been little effort to attempt this, so more research is still needed. Textile-based methods need to be integrated into shirts to be feasible for everyday use and should be washing machine safe. Solutions involving neck bands or collars would likely have low compliance in the real world, though may be accurate in a lab setting. RIP has promising results detecting swallowing, but no research has been done to estimate the intake volume. It has also not been tested for compliance when integrated into shirts. All reviewed RIP studies were only tested in lab settings with small subject groups.

### 8.2. Surfaces

Sensors embedded in surfaces are much less common and less investigated. The studies that used an entire table as the surface are mainly focused on determining food intake while the studies that used coasters focused on determining drink intake. It is more feasible to determine drinking actions than eating because the entire container must be lifted up and set back down on the surface every time. This is an action that is simpler to detect compared to differentiating fork and spoon use. However, without other information to make the system contextually aware in the environment, any object can be placed or removed from the surface which may result in many false alarms.

### 8.3. Vision and Environmental Based

In vision-based approaches, privacy is a major concern. Some researchers combat this by only capturing the region of interest and not the faces. However, this creates another challenge for how to identify this region. There exist studies that used segmentation algorithms to remove faces from the captured data [102]. Some researchers rely on the depth data obtained from RGB-D cameras to resolve these issues. Overall, the accuracy of the algorithms performed on the data is limited to the resolution of the camera. Additionally, vision-based methods also performed poorly when there is low lighting and require high processing power and storage of the data. The review of the literature also showed that approaches using wearable cameras often lead to a large loss of data and are usually not automated. The majority of vision-based approaches used deep learning to classify images, relied on static images, and did not test the detection performance in real time. The classification accuracy was often above 90% when using deep learning models, which is promising. Using environmental methods such as radar would remove the concerns around privacy and the need for a well-lit environment. However, very few papers used radar or RFID and more research is needed in this field. Additionally, there was no research done using other types of environmental approaches such as LiDAR or thermal IR. None of these vision or environmental-based approaches estimate intake amount and they only focus on intake detection. 

### 8.4. Smart Container

Placing sensors in the water vessel has yielded effective and accurate results when determining the intake volume consumed. However, there are still some limitations in the commercial smart bottles. For instance, these bottles are targeted toward active young adults and are mostly large, heavy and not tailored to seniors. Most of the commercial bottles also rely on displaying the information in a mobile app, which can be too complicated for a senior. Some bottles display the information on a screen directly on the bottle, but often the data is not stored and available for the users. Although several smart bottles and cups are mentioned in research, they were not available to purchase in Canada, including the Droplet, Ozmo, Smart CUP, and DrinkUp.

In the literature reviewed, the majority of techniques had an accuracy above 90% for detecting drinking volume. This review also showed that using solely an inertial system provided lower results compared to fusing the inertial data with another method to detect liquid level or weight. Sensors embedded in containers have their own limitations. For example, the user needs to drink only from a specific container. Several studies investigated ultrasonic sensors to determine liquid volume in a container, but very few tested the accuracy of the systems. Thus, there is a need to create an accurate smart bottle that is inexpensive, light, and easy to use especially for an older population, and that is also commercially available. 

### 8.5. Fusion

Most of the fusion techniques reviewed involved a wrist accelerometer and one other technology. This is likely due to the good accuracy but high false positive rates that a single accelerometer provides. Combining the acceleration data with other devices could create more accurate systems. This is an area that can be explored further to increase the accuracy of systems and reduce false positives. Additionally, many of the fusion studies found were only preliminary pilot studies with no results or were conducted on a small sample size. Fusing sensors is promising to obtain an accurate system, but it is essential that the combination is not cumbersome to use. 

### 8.6. Real-World Datasets

Of the reviewed studies, only four systems were tested in free living conditions, and seven were tested in semi-free living (non-scripted) conditions, such as allowing the participant to act freely during a shorter period of time like during a meal. None of the textile studies or vision-based approaches tested the systems in free living conditions. Many of the surface-based studies used semi-free living conditions as they tested the system during an entire meal time. The majority of the free living studies used wearables (3 out of 4). As expected, the free living studies provided lower system performance compared to laboratory conditions. Two out of four free-living studies provided real-time validation [47,125]. The longest study was conducted for 5 months in a nursing home [125] to validate if volume intake increased when the patients were prompted by nurses. Table 6 summarizes the performance for all free living studies reviewed in this paper.

## 9. Conclusions

This paper provides a review for technological fluid intake monitoring systems, focusing on their applicability for elderly people. Although many papers mentioned the impact their systems may have on the elderly population, very few were tested on this age group. Many of the devices reviewed did not estimate volume intake and only focused on intake detection. Only embedded surfaces and drinking containers were able to estimate volume level, accurately. Sensors in fluid containers almost always required the user to place the device on a surface for a drink to be recorded, as do sensors in the surfaces. Additionally, the user must drink from a specific container or in a specific location. Vision-based approaches had high accuracy of image detection, but they cannot estimate volume intake, and may be subject to occlusion or poor performance under various light conditions. They also only work when the user is at home. Many studies included depth cameras to avoid potential privacy concerns. There are a lack of studies using other environmental approaches such as radar, infrared, RFID or LiDAR, which also preserve privacy. Wearable technology has been studied extensively for activity recognition including nutrition-intake recognition. Wrist wearables are versatile in all environments but are subject to a high false positive rate when used alone. Determining intake volume from wrist acceleration alone was investigated but yielded low accuracy. Textile solutions are promising, but more investigation is needed to make a textile that is practical and economical for everyday use. Although there are many promising studies in the literature with high accuracy that could monitor senior hydration level, most of these studies do not reach the commercial market. It is important to create products for the end user as there is no widely, commercially available product that aids monitoring and tracking senior liquid intake automatically.

## Figures and Tables

**Table 1:** Summary of the top seven wearable literature, where #Sen. corresponds to the number of sensors used and #Sub. corresponds to the number of subjects in the study. The system accuracy denotes the overall average accuracy when classifying all actions. The drink detection accuracy shows the accuracy for detecting the drinking action only. The same is applied for the F1-scores.

Ref.	#Sen.	Method	#Sub	System Accuracy (%)	Drinking Detection Accuracy (%)	System F1-Score (%)	Drinking Detection F1-Score (%)	Null Class
[46]	2	Binary CNN ^1^ LSTM ^2^	41	95.7	-	96.5	-	√
				81.4		85.5		
[52]	1	5-class RNN ^3^ + LSTM ^2^	NA	99.6	100	99.2	100	×
[54]	1	Binary RF ^4^	6	97.4	97.4	96.7	95.3	√
		3-class ANN ^5^		98.2	99	95.3	93.3	
		5-class ANN ^6^		97.8	98.6	87.2	90.9	
[59]	1	2-stage CRF ^7^: 8-class	70	-	-	60	85.5	√
		3-class				81.1	93.4	
[60]	1	Binary Adaboost	20	94.4		96.2	-	√
		5-class RF ^4^		-	-	91	95	
[61]	5	9-class SVM ^7^	20	91.8	-	91.1	-	×
	2			89		88.4	93.4	
[62]	3	3-stage SVM ^7^ + HMM ^8^	14	-	-	87.2	-	√

^1^ Convolutional Neural Network, ^2^ Long Short Term memory, ^3^ Recurrent Neural Network, ^4^ Random Forest, ^5^ Artificial Neural Network, ^6^ Conditional Random Fields, ^7^ Support Vector Machine, ^8^ Hidden Markov Model

**Table 2:** Summary of surface embedded literature. The drinking detection accuracy shows the classification accuracy for detecting drinking action only, while the system accuracy is the average classification accuracy considering all classes. The weight error/accuracy shows the performance for identifying the volume intake.

Ref.	#Sen	Method	#Sub	System Accuracy (%)	Drinking Detection Accuracy (%)	Weight Error/ Accuracy	Limitations
[87]	9+	Rule-based, template matching	3	80	-	82.62% accuracy	Small sample size, all objects need RFID
[89]	1264	DT ^1^, 7-class No LOSO ^2^	5	91	99	16% RMSE	Low weight accuracy
		With LOSO ^2^		76	99		
[91]	1	Segmentation and thresholding	271	39% of bites are undetected	39% of drink sips undetected	-	Many false positives and undetected intakes
[92]	8	Comparing against acoustic neck microphone	2	-	-	<9 g error	Small sample size

^1^ Decision Tree, ^2^ Leave-one-subject-out

**Table 3:** Summary of vision- and environmental-based literature.

Ref.	#Sen.	Method	#Sub	System Accuracy/ Precision (%)	Drinking Detection Accuracy (%)	Null Class
[101]	1	ANN ^1^	33	98.3	-	×
[104]	1	3D CNN ^2^, 13 classes	1950 videos	96.4	92	√
[102]	4	Fuzzy vector quantization, LDA ^3^ 3 class	4	93.3	100	√
[105] *	2	kNN ^4^ 4 class	2	89.13	100	√
		kNN ^4^ 6 class		95.4	93.1	
		kNN ^4^ 5 class		98.7	96.88	

* This paper only reported the precision values and the accuracy values were not reported by the authors. ^1^ Artificial Neural Network, ^2^ Convolutional Neural Network, ^3^ Linear Discriminant Analysis, ^4^ k-Nearest Neighbours. Where #Sen. corresponds to the number of sensors used and #Sub. corresponds to the number of subjects in the study.

**Table 4:** Summary of top eight smart containers literature.

Ref.	Technology	#Sen.	#Sub	System Accuracy (%)	Weight Error
[120]	IMU	1	7	99	25% volume
[125]	Strain gauge + IMU	2	15	-	2 mL
[126]	Capacitance	20	1	-	3–6%
[127]	Conductive electrodes + IMU	6	15	94.33	-
[128]	Metal tag + WiFi	3	-	90	-
[129]	Vibration transducer + WiFi	1	6 liquids, 3 containers	>97	<10% liquid level
[147]	IMU + ultrasound, humidity/temperature sensor + pH + turbidity sensor	6	6	-	-
[131]	Water flow sensor	1	Unknown	-	8 mL, 2%

Where #Sen. corresponds to the number of sensors used and #Sub. corresponds to the number of subjects in the study.

**Table 5:** Summary of commercial smart bottles.

Product Name	Price (USD)	Pros	Cons	Size (oz)
Hidrate Spark 3	$59.95	Clinically validated, Offline glow reminders, Plastic—Light, Saves data locally, sync later	Not rechargeable, No API, Large size	20
Hidrate Spark Steel	$64.99	Clinically validated, Rechargeable, Offline glow reminders, Allows ice, Saves data locally, sync later	Hand wash only, No hot drinks, 10–14 day battery, No API, Steel—Heavy	17/21
H2OPal	$99.99	API available, Compatible with any bottle of same size, Dishwasher safe, Saves data locally, sync later, Hot liquid allowed	Needs setup, Not rechargeable, No offline reminders	18.6
Ozmo Active/ Java+	$69.99	Differentiates water and coffee, Java + regulates temperature, Real-time sync in app, Rechargeable, LED to indicate hydration goals, Offline vibration reminders	Hand wash only, No API	16
Thermos Smart lid	$42.35	Temperature sensor, Rechargeable, Stores locally for up to 1 week, Plastic—Light	No hot liquids, Must be upright to record, Large size	24
DrinKup	$69	Shows amount and temperature, Determines whether water is stale, Allows ice, Rechargeable, Stores locally, Simple, subtle design	Not available, limited information	17
HydraCoach 2.0	$27.94	Allows ice, Dishwasher safe, Results directly on bottle	Low-intensity sips may not register, Offline use only, No data transfer, No hot drinks	22
Droplet	$47.53	Designed for elderly (light, ergonomic), Looks like normal cup/mug, Compatible base, Light, Voice reminders on bottle, Dishwasher safe	Offline, No access to data	9.5–11.2

**Table 6:** Summary of all free-living studies reviewed in this paper. The mL indicates weight error accuracy, not an F1-score.

Ref.	Technology	#Sub	Duration of Data	F1-Score from Lab	F1-Score from Free Living Conditions
[47]	Wearable	12 lab, 5 free living	-	97%	85%
[56]	Wearable	7 free living	35 days	-	75.6%
[59]	Wearable	70 total, 8 free living	24 h	85.5%	68.5%
[125]	Smart Container	15 free living	5 months	-	2 mL
